# ABO blood type and clinical characteristics of patients with ulcerative colitis: A hospital-based study in central Taiwan

**DOI:** 10.1371/journal.pone.0260018

**Published:** 2022-02-03

**Authors:** Hsiang-Chun Lai, Jen-Wei Chou, Yi-Hua Wu, Po-Ju Huang, Ken-Sheng Cheng, Tsung-Wei Chen

**Affiliations:** 1 Department of Chinese Medicine, China Medical University Hospital, Taichung, Taiwan; 2 Center for Digestive Medicine, Department of Internal Medicine, China Medical University Hospital, Taichung, Taiwan; 3 The Taiwan Society of Inflammatory Bowel Disease (TSIBD), Taipei, Taiwan; 4 Taiwan Association for the Study of Small Intestinal Diseases (TASSID), Touyuan, Taiwan; 5 Department of Pathology, Asia University Hospital, Taichung, Taiwan; Changhua Christian Healthcare System: Changhua Christian Hospital, TAIWAN

## Abstract

**Background:**

The variations in ABO blood groups are reported to be associated with multiple disorders, including ulcerative colitis (UC). We aimed to investigate the distribution of ABO blood groups in UC patients and explore its impact on disease severity.

**Methods:**

We retrospectively collected 129 UC patients diagnosed at our hospital between January 2000 and November 2019. Clinical characteristics, ABO blood groups, and operation rates were analyzed.

**Results:**

The mean diagnostic age of patients was 38.97 years. Males accounted for the majority of all patients (62.8%). Of 129 patients, 43 (33.3%) were blood type O, 41 (31.8%) were blood type A, 38 (29.5%) were blood type B, and 7 (5.4%) were blood type AB. Although our patients had higher ratio of blood type A comparing our general population, there was no statistically significant association of ABO blood types distribution between these two groups (p = 0.1906). In the subgroup analysis, there were no significant difference of disease locations and operation rates between different ABO blood groups. Furthermore, blood type A patients had higher serum hemoglobin (Hb) levels compared to blood type O patients (13.31 g/dL vs. 12.30 g/dL, p = 0.0347). Blood type A patients had lower serum erythrocyte sedimentation rate (ESR) levels compared to blood type O patients (12.46 mm/hour vs. 21.5 mm/hour, p = 0.0288). Blood type O had higher serum ESR levels compared to non-O groups (p = 0.0228). In the ABO blood groups and mean diagnostic age (≤ 40 years or > 40 years), there were no statistically significant difference between these two age groups, p = 0.5515.

**Conclusions:**

Our results showed ABO blood groups are not associated with UC in spite of a higher ratio of blood type A in our patients. Blood type O patients had higher serum ESR levels; however, blood type A patients had higher Hb levels.

## Introduction

Inflammatory bowel disease (IBD), including ulcerative colitis (UC) and Crohn’s disease (CD), is a complex disease with interaction of genetics, the environment and the gut microbiota [1]. UC is a chronic and relapsing disease of the gastrointestinal tract with unclear etiology. Risk factors such as age, ethnicity, family history, smoking, appendectomy, microbiota change, diet and urbanization lifestyle were mentioned for UC in some studies in the literature [1–3]. The reported incidence rates of IBD are higher in North America and Western Europe [4]. On the contrary, the incidence rates of IBD are still low but increasing in East Asia, including Taiwan [4]. Thus, IBD has become an important issue to our clinical practice. Recently, the variations in ABO blood groups are mentioned to be associated with multiple disease such as coronary artery disease, infection susceptibility and hepatocellular carcinoma [5–7]. The presence of blood type A antigen has been reported in fetal mucosa and adenocarcinomas of distal colon [5]. However, only a few studies have discussed on the association between ABO system and UC in 1960s, but those results were controversial [6–8]. In recent years, researches in discussing the association between ABO blood groups and UC were scarce, especially in Asia. Therefore, our current study was to investigate the distribution of ABO blood groups in patients with UC and its correlation to patient’s clinical characteristics in Taiwan.

## Patients and methods

### 1. Study population

We retrospectively searched the database of chart records from January 2000 through November 2019 in China Medical University Hospital (CMUH), a medical center in central Taiwan. We identified our UC patients by the International Classification of Disease (2001 version) for disease coding, UC as 556.XX in CMUH chart records. We included patients who were diagnosed as UC with ABO blood type records. The diagnostic criteria for UC was based on evaluation of clinical, endoscopic, and pathologic findings, and excluded those with an infectious etiology. The ABO blood type was confirmed by either laboratory reports or self-reports of UC patients. The follow-up duration of the patients initiated at the time of diagnosis or the first-time visit to our clinic and ended at the last time recorded in the chart. We retrospectively collected the clinical characteristics of UC patients, including gender, age at diagnosis, ABO blood groups, baseline laboratory tests such as hemoglobin (Hb) levels, serum albumin levels, serum C-reactive protein (CRP) levels, serum erythrocyte sedimentation rate (ESR) levels, disease phenotypes, and operation rates. The disease phenotype was based on Montreal classification as extent 1 for ulcerative proctitis, extent 2 for left sided UC and extent 3 for extensive UC (pancolitis).

### 2. Statistical methods

With respect to statistical methods, descriptive statistics were presented in the form of mean (standard deviation) for continuous variables, and as frequency and proportion (%) for categorical variables. We compared the characteristics between two groups by using either a two-sample t-test or Wilcoxon rank-sum test for continuous variables, and a Chi-square test or Fisher’s exact test for categorical variables. We compared the characteristics across four blood types (O, A, B, AB) using ANOVA test for continuous variables and Fisher’s exact test for categorical variables. We conducted pairwise comparison across four blood types using Tukey’s test. We compared the blood type distribution of our patients and reference general population with goodness of fit test. All statistical analyses were performed using SAS version 9.4 (SAS Institute Inc., Cary, NC, USA) and R version 3.5.1 (R Foundation for Statistical Computing, Vienna, Austria). The significance level was set at 0.05, and all tests were two-tailed.

### 3. Ethics statements

All methods were carried out in accordance with relevant guidelines and regulations. Informed consent was obtained from all subjects. The methods section that the research was carried out in accordance with the Helsinki Declaration. This study was approved by the institutional review board of the Research Ethic Committee of China Medical University Hospital, in Taiwan (CMUH107-REC1-139).

## Results

A total of 129 patients with UC were enrolled into our current study. The clinical characteristics of all patients are shown in Table 1. With respect to the sex distribution, we included 81 male patients and found out males accounted for the majority (81/129, 62.79%) of all patients. The mean diagnostic age of our UC patients was 38.97 ± 14.46 years (ranging from 9–83 years). In the analysis of baseline laboratory tests, we found Hb levels were 12.79 ± 2.41 g/dL, serum albumin levels were 4.24 ± 0.59 g/dL, serum CRP levels were 1.51 ± 3.83 mg/dL, and serum ESR levels were 16.11 ± 16.49 mm/hour, respectively. Moreover, the baseline Mayo scores of our all UC patients were 8.28 ± 2.45 points. In the analysis of disease phenotypes of UC according to the Montreal classification, we found the incidence rates of Extent 1 (E1), Extent (E2), and Extent 3 (E3) at diagnosis were 22.48%, 37.98%, and 39.53%, respectively. Extensive UC accounted for the majority of our all UC patients. The operation rate in our all UC patients was 5.43% (7/129).

**Table 1:** Clinical characteristics of patients with ulcerative colitis.

Characteristics	Total UC N = 129
Male gender, n (%)	81 (62.79)
Diagnostic age, years, mean (SD)	38.97 (14.46)
Baseline albumin (g/dL), mean (SD)	4.24 (0.59)
Baseline Hb (g/dL), mean (SD)	12.79 (2.41)
Baseline CRP (mg/dL), mean (SD)	1.51 (3.83)
Baseline ESR (mm/hour), mean (SD)	16.11 (16.49)
Baseline Mayo score, mean (SD)	8.28 (2.45)
Disease location	
Extent 1, n (%)	29 (22.48)
Extent 2, n (%)	49 (37.98)
Extent 3, n (%)	51 (39.53)
Operation rate, n (%)	7 (5.43)

Abbreviations: CRP, C-reactive protein; ESR, erythrocyte sedimentation rate; Hb, hemoglobin; UC, ulcerative colitis.

The distribution ratios of ABO blood groups in our all UC patients and the general population are shown in Table 2. With respect to the ABO blood groups of 129 UC patients, 43 patients (33.33%) were blood type O, 41 (31.78%) were blood type A, 38 (29.46%) were blood type B, and the remainders 7 (5.43%) were blood type AB. In a report by Sun et al., they concluded the distribution ratios of ABO blood groups in the general population of Taiwanese were 42.9%, 24.3%, 26.8% and 6.0% for O, A, B and AB, respectively [20]. From our current study, we observed a trend toward lower frequency of the blood type O in our UC patients compared to the control group of general population (33.3% vs. 42.9%). We also found a trend toward higher frequency of the blood type A in our UC patients compared to the control group of general population (31.78% vs. 24.3%). Moreover, we found similar ratios of the blood B and blood type AB in our UC patients compared to the control cases of general population (blood type B: 29.46% vs. 26.8%; blood type AB: 5.43% vs. 6%). However, there was no statistically significant association between ABO blood groups and UC risk compared to the control cases of general population of Taiwanese (p = 0.1906).

**Table 2:** ABO blood types distribution of patients with ulcerative colitis.

Blood type	Our study	General population of Taiwanese*
O type	43 (33.33%)	42.9%
A type	41 (31.78%)	24.3%
B type	38 (29.46%)	26.8%
AB type	7 (5.43%)	6%

X2 = 4.7555 (p = 0.1906) with goodness of fit test.

* Reference: Wenjie Sun, et al. Cancer Epidemiol. 2015;39:150–156.

In the subgroup analysis of the association of different ABO blood types and baseline laboratory tests are shown in Table 3. From the results of our current study, we found blood type A UC patients had higher Hb levels compared to blood type O patients with a statistically significant difference (blood type A: 13.31 g/dL vs. blood type O: 12.30 g/dL, p = 0.0347). Blood type B UC patients had lower serum ESR levels compared to blood type O patients with a statistically significant difference (blood type B: 12.46 mm/hour vs. blood type O: 21.5 mm/hour, p = 0.0288). Moreover, blood type O UC patients had the higher serum ESR levels compared to non-O groups with a statistically significant difference (p = 0.0228). Blood type O UC patients also had a trend of higher E2 rate compared to non-O group (46.51% vs. 33.72%, p = 0.3503) but without significance. Blood type B UC patients had a trend of the highest E3 rate (47.37%). Blood type AB UC patients had a trend of the lowest E3 rate (14.29%). Moreover, blood type A UC patients had the highest operation rate (7.32%), followed by blood type O (6.98%), blood type B (2.63%) and blood type AB (0%). Nevertheless, there was no statistically significant difference of gender distribution, diagnostic mean age, baseline serum albumin levels, baseline serum CRP levels, baseline Mayo scores, disease phenotypes and operations rates between different ABO blood groups and four-group comparison (S1 Table).

**Table 3:** Blood type subgroup analysis of clinical characteristics of patients with ulcerative colitis.

	Blood type O		Non-blood type O			p value^a^	p value^d^
	(N = 43)		(N = 86)			p value^b^	
						p value^c^	
Characteristics		All	Blood type A	Blood type B	Blood type AB		
		(N = 86)	(N = 41)	(N = 38)	(N = 7)		
Male gender, n (%)	29 (67.44)	52 (60.47)	27 (65.85)	21 (55.26)	4 (57.14)	0.8773^a^	0.4396
						0.2604^b^	
						0.6768^c^	
Diagnostic age, years, mean (SD)	38.56 (15.03)	39.17 (14.26)	41.29 (12.94)	38.16 (15.20)	32.29 (15.65)	0.3472^a^	0.8376
						0.8350^b^	
						0.2514^c^	
Baseline albumin (g/dL), mean (SD)	4.23 (0.65)	4.25 (0.57)	4.36 (0.47)	4.13 (0.65)	N^e^	0.5894^a^	0.8320
						0.4072^b^	
						N^e^	
Baseline Hb (g/dL), mean (SD)	12.30 (2.49)	13.04 (2.34)	13.31 (2.07)	12.81 (2.62)	12.68 (2.41)	**0.0347** ^a^	0.0688
						0.2842^b^	
						0.7274^c^	
Baseline CRP (mg/dL), mean (SD)	2.48 (5.42)	0.98 (2.49)	1.43 (3.47)	0.55 (0.69)	0.57 (0.78)	0.2948^a^	0.0882
						0.2235^b^	
						0.5600^c^	
Baseline ESR (mm/hour), mean (SD)	21.5 (20.99)	13.2 (12.68)	13.43 (13.45)	12.46 (11.96)	17.75 (14.36)	0.0564^a^	**0.0228**
						**0.0288** ^b^	
						0.9349^c^	
Baseline Mayo score, mean (SD)	8.27 (2.56)	8.29 (2.41)	8.32 (2.72)	8.34 (2.02)	7.86 (2.79)	0.8509^a^	0.8342
						0.6859^b^	
						0.5620^c^	
Disease location						0.5417^a^	0.3503
						0.2448^b^	
						0.3880^c^	
Extent 1, n (%)	9 (20.93)	20 (23.26)	8 (19.51)	9 (23.68)	3 (42.86)		
Extent 2, n (%)	20 (46.51)	29 (33.72)	15 (36.59)	11 (28.95)	3 (42.86)		
Extent 3, n (%)	13 (32.56)	37 (43.02)	18 (43.9)	18 (47.37)	1 (14.29)		
Operation rate, n (%)	3 (6.98)	4 (4.65)	3 (7.32)	1 (2.63)	0 (0)	1.0000^a^^a^	0.6854
						0.6184^b^	
						1.0000^c^	

Abbreviations: CRP, C-reactive protein; ESR, erythrocyte sedimentation rate; Hb, hemoglobin; UC, ulcerative colitis.

^a^ compare blood type O and blood type A.

^b^ compare blood type O and blood type B.

^c^ compare blood type O and blood type AB.

^d^ compare blood type O and blood type non-O.

^e^ can’t calculate due to small sample size.

The clinical characteristics of different diagnostic mean age (≤ 40 years old or > 40 years old) with ABO blood groups are shown in S2 Table. We yielded 68 UC patients were ≤ 40 years old and 61 UC patients were > 40 years old. There were 44 male gender patients (44/129, 64.71%) in younger diagnostic age group and 37 male gender patients (60.66%) in older diagnostic age group. In younger diagnostic age group, we found the distribution ratios of blood type O, A, B, and AB were 22 patients (32.35%), 19 patients (27.94%), 22 patients (32.35%) and 5 patients (7.35%). In older diagnostic age group, we found the distribution ratios of blood type O, A, B, and AB were 21 patients (34.43%), 22 patients (36.07%), 16 patients (26.23%) and 2 patients (3.28%), respectively. Thus, we found our UC patients with younger diagnostic age group had more blood type B compared to the control cases of general population. In contrast, UC patients with older diagnostic age had more blood type A compared to the control cases of general population. However, there were no statistically significant difference between these two diagnostic age groups (p = 0.5515).

## Discussion

In a literature review, we found Buckwalter et al. firstly analyzed the correlation of UC patients and ABO blood type distribution in 1956 [9]. They enrolled 184 UC patients and reported the distribution ratios of blood type O, A, B, AB were 43.5%, 44.6%, 8.1% and 3.8%, respectively. However, they didn’t find a significant difference between the UC patients and control cases. In a study of 317 UC patients reported by Smith et al., they found the distribution ratios of blood type O, A, B, AB were 46.37%, 39.12%, 10.41% and 4.1%, respectively [10]. They also concluded no relation between the ABO blood types and UC patients. In a study reported by Winstone et al., they did not find UC to be correlated with the ABO blood groups, secretor status, or the rhesus (Rh) factor [11]. Moreover, Boyd et al. first reported 185 UC patients with colectomy and found their patients had higher homozygous CC Rh type [12]. However, they also concluded that there was no correlation between the UC patients and ABO blood groups, MN or secretor type. Thayer and Bove demonstrated a study of ABO blood groups in 170 UC patients and also had in agreement with previous reports [6]. Recently, some new researches of the association between ABO blood groups and IBD were discussed in the literature. Forni et al. reported the non-O blood group and the non-secretor status were associated with higher risk of stricture or penetrating type of CD, while no correlation with ABO variants [13]. Yu et al. also demonstrated CD patients with blood type AB had a better response to infliximab, while those with blood type A tend to have a risk of losing response to infliximab [14]. They also concluded ABO blood groups were not associated with the prevalence of CD patients.

However, clinical researches in discussing the association of ABO blood groups and IBD patients were scarce in Asian countries. Recently, Ye et al. conducted a study of association of fucosyltransferase 2 (FUT2) and ABO blood groups with CD in Koreans [15]. They concluded the O blood group and FUT2 secretor status were protective factors for CD patients. To the best of our knowledge, there was no associated report of ABO blood groups with IBD in Taiwan. Thus, we conducted the first study of ABO blood groups associated with UC patients in Taiwan. From the results of our current study, we found UC patients had a trend of less O blood type and more A and B blood types compared to those in control cases of general population in Taiwan. Our present study also showed less O blood type and more blood types A and B compared to the general population of Taiwan, which is similar with the previous study reported by Ye et al. in Korea. Our results showed a male predominance in all UC patients, which was similar to the previous Asian report [3]. In the disease phenotypes of UC patients, most Western studies showed proctitis in 30–60%, left-sided colitis in 16–40%, and extensive colitis in 18–35%. We reported more extensive colitis and left-sided UC patients and less proctitis, which was similar to our previous study [16]. There was no statistically significant difference of disease phenotypes and baseline laboratory tests between groups due to limited case numbers. And we found there were lack of reports about disease phenotypes and laboratory tests, which served as a reference. Among the seven operation times, three patients underwent colectomy, three patients underwent fistula intervention and one underwent endoscopic submucosal dissection due to esophageal cancer. The operation rate was the highest in blood type A group (7.32%) and followed by blood type O group (6.98%), while more cases were needed to meet a conclusion. We hypothesized the trend of ABO blood types distribution in UC patients was contributed by the surface glycoprotein susceptibility of gut microbiota. As we know, microbiota change plays a role in the occurrence of IBD [4, 17]. Blood group antigen expression interfere host susceptibility to infection such as microorganisms, parasites, and viruses. There would be symbiotic relationship between blood group expression and gastrointestinal microbiome and affect IBD occurrence [18, 19]. Further basic researches are needed to identify the pathophysiological mechanism.

Nevertheless, our current study has several limitations. First, although our hospital is the biggest tertiary hospital in central Taiwan, our hospital-based study had a relatively small sample size and was retrospective design. There may have been a selection bias. Thus, more prospective population-based studies at the national level are required in order to identify the correlation between ABO blood groups and UC patients. Second, we lacked the proper control group for analysis. Thus, we applied a large-sample sized cohort study of healthy individuals in Taiwan as a control group [20]. Third, we only reported the association of ABO blood groups and UC patients but lacked the comprehensive blood system analysis such as Rh system, MN system and FUT2 data in our current study. Therefore, more detailed studies would apply in the future.

## Conclusions

Our current study demonstrated the first report of association of ABO blood groups and UC patients in Taiwan. We found UC patients had a trend of less blood type O and more blood types A and B compared to the control cases of our general population. Furthermore. UC patients with young diagnostic age showed more blood type B; in contrast, UC patients with older diagnostic age showed more blood type A. However, these above results didn’t reach a significant difference in comparing to the control cases of general population, In the subgroup analysis, blood type O UC patients had higher serum ESR levels and blood type B UC patients had lower serum ESR levels with a significance. UC patients with blood type A had higher Hb levels with significance. Our finding might suggest that blood type O presented less prevalence, more E2 type and higher baseline serum ESR levels. We hypothesis the trend of ABO distribution in UC patients was contributed by the surface glycoprotein susceptibility of gut microbiota. However, more prospective population-based studies at the national level and basic researches are needed to identify the blood type distribution of UC patients and its physiopathological mechanism.

## Supporting information

S1 Table Blood type subgroup analysis of clinical characteristics of patients with ulcerative colitis. (DOCX)

S2 Table Clinical characteristics of early diagnosed and late diagnosed patients with ulcerative colitis. (DOCX)

S1 Data UC of blood type. (XLSX)

## Abbreviations

CD: Crohn’s disease
CRP: C-reactive protein
ESR: Erythrocyte sedimentation rate
FUT2: fucosyltransferase 2
Hb: Hemoglobin
IBD: Inflammatory bowel disease
Rh: rhesus
UC: Ulcerative colitis
