# Supplementary material for: ABO blood type and clinical characteristics of patients with ulcerative colitis: A hospital-based study in central Taiwan
Source: PLoS One. 2022 Feb 3;17(2):e0260018. doi: 10.1371/journal.pone.0260018 (PMC8812853; doi:10.1371/journal.pone.0260018)
Supplement: S1 Table — (DOCX) [file pone.0260018.s001.docx]

Supplementary Table 1. Blood type subgroup analysis of clinical characteristics of patients with ulcerative colitis

|  | Blood type O  (N = 43) | | Non-blood type O  (N = 86) | | | p value^a^  p value^b^  p value^c^ | O vs non-O comparison p value^d^ | Four-group comparison  p value^e^ |
| --- | --- | --- | --- | --- | --- | --- | --- | --- |
| Characteristics |  | All  (N = 86) | Blood type A  (N = 41) | Blood type B  (N = 38) | Blood type AB  (N = 7) |  |  |  |
| Male gender, n (% ) | 29 (67.44) | 52 (60.47) | 27 (65.85) | 21 (55.26) | 4 (57.14) | 0.877^a^  0.260^b^  0.677^c^ | 0.4396 | 0.666 |
| Diagnostic age, years,  mean (SD) | 38.56 (15.03) | 39.17 (14.26) | 41.29 (12.94) | 38.16 (15.20) | 32.29 (15.65) | 0.822^a^  0.999^b^  0.712^c^ | 0.8376 | 0.443 |
| Baseline albumin (g/dL), mean (SD) | 4.23 (0.65) | 4.25 (0.57) | 4.36 (0.47) | 4.13 (0.65) | N^e^ | 0.817^a^  0.915^b^  0.998^c^ | 0.8320 | 0.510 |
| Baseline Hb (g/dL),  mean (SD) | 12.30 (2.49) | 13.04 (2.34) | 13.31 (2.07) | 12.81 (2.62) | 12.68 (2.41) | 0.236^a^  0.781^b^  0.982^c^ | 0.0688 | 0.312 |
| Baseline CRP (mg/dL), mean (SD) | 2.48  (5.42) | 0.98  (2.49) | 1.43  (3.47) | 0.55  (0.69) | 0.57  (0.78) | 0.653^a^  0.168^b^  0.715^c^ | 0.0882 | 0.203 |
| Baseline ESR (mm/hour), mean (SD) | 21.5  (20.99) | 13.2  (12.68) | 13.43  (13.45) | 12.46  (11.96) | 17.75  (14.36) | 0.142^a^  0.080^b^  0.971^c^ | **0.0228** | 0.071 |
| Baseline Mayo score,  mean (SD) | 8.27  (2.56) | 8.29  (2.41) | 8.32  (2.72) | 8.34  (2.02) | 7.86  (2.79) | 0.999^a^  0.999^b^  0.977^c^ | 0.8342 | 0.971 |
| Disease location |  |  |  |  |  |  | 0.3503 | 0.453 |
| Extent 1, n (%) | 9 (20.93) | 20 (23.26) | 8 (19.51) | 9 (23.68) | 3 (42.86) |  |  |  |
| Extent 2, n (%) | 20 (46.51) | 29 (33.72) | 15 (36.59) | 11 (28.95) | 3 (42.86) |  |  |  |
| Extent 3, n (%) | 14 (32.56) | 37 (43.02) | 18 (43.9) | 18 (47.37) | 1 (14.29) |  |  |  |
| Operation rate, n (%) | 3 (6.98) | 4 (4.65) | 3 (7.32) | 1 (2.63) | 0 (0) | 1.0000^a^  0.6184^b^  1.0000^c^ | 0.6854 | 0.799 |

Abbreviations: CRP, C-reactive protein; ESR, erythrocyte sedimentation rate; Hb, hemoglobin; UC, ulcerative colitis

^a^ Pairwise comparison between blood type O and blood type A using Tukey’s test.

^b^ Pairwise comparison between blood type O and blood type B using Tukey’s test.

^c^ Pairwise comparison between blood type O and blood type AB using Tukey’s test.

^d^ Compare blood type O and blood type non-O using Wilcoxon rank-sum test for continuous variable and Chi-square test for categorical variables.

^e^ Four-group comparison of blood type O, A, B, and AB using ANOVA for continuous variables and Fisher exact test for categorical variables.
