# Supplementary material for: ABO blood type and clinical characteristics of patients with ulcerative colitis: A hospital-based study in central Taiwan
Source: PLoS One. 2022 Feb 3;17(2):e0260018. doi: 10.1371/journal.pone.0260018 (PMC8812853; doi:10.1371/journal.pone.0260018)
Supplement: S2 Table — (DOCX) [file pone.0260018.s002.docx]

Supplementary Table 2. Clinical characteristics of early diagnosed and late diagnosed patients with ulcerative colitis

| Characteristics | Diagnostic age ≤ 40 years  N = 68 | Diagnostic age > 40 years  N = 61 | p-value |
| --- | --- | --- | --- |
| Male gender, n (%) | 44 (64.71) | 37 (60.66) | 0.6347 |
| Diagnostic age, years, mean (SD) | 27.50 (6.76) | 51.75 (8.94) | **< 0.0001** |
| Age at biological agent, years, mean (SD) | 33.64 (10.76) | 54.57 (8.82) | **< 0.0001** |
| Blood type |  |  | 0.5515 |
| O type, n (%) | 22 (32.35) | 21 (34.43) |  |
| A type, n (%) | 19 (27.94) | 22 (36.07) |  |
| B type, n (%) | 22 (32.35) | 16 (26.23) |  |
| AB type, n (%) | 5 (7.35) | 2 (3.28) |  |
| Baseline albumin (g/dL), mean (SD) | 4.31 (0.66) | 4.18 (0.52) | 0.0662 |
| Baseline Hb (g/dL), mean (SD) | 12.83 (2.62) | 12.76 (2.18) | 0.5468 |
| Baseline CRP (mg/dL), mean (SD) | 1.83 (4.74) | 1.20 (2.72) | 0.6627 |
| Baseline ESR (mm/hour), mean (SD) | 14.97 (14.76) | 17.30 (18.16) | 0.2077 |
| Baseline Mayo score, mean (SD) | 8.33 (2.24) | 8.23 (2.68) | 0.8331 |
| Disease location |  |  | 0.5565 |
| Extent 1, n (%) | 17 (25) | 12 (19.67) |  |
| Extent 2, n (%) | 27 (39.71) | 22 (36.07) |  |
| Extent 3, n (%) | 24 (35.29) | 27 (44.26) |  |
| Operation rate, n (%) | 4 (5.88) | 3 (4.92) | 1.0000 |

Abbreviations: CRP, C-reactive protein; ESR, erythrocyte sedimentation rate; Hb, hemoglobin
